# Not All Genes Are Equal; Shortage of Histones Affects Some Genes More Than Others

**DOI:** 10.1371/journal.pbio.1001098

**Published:** 2011-06-28

**Authors:** Robin Mejia

**Affiliations:** Freelance Science Writer, Albany, California, United States of America

**Figure:**
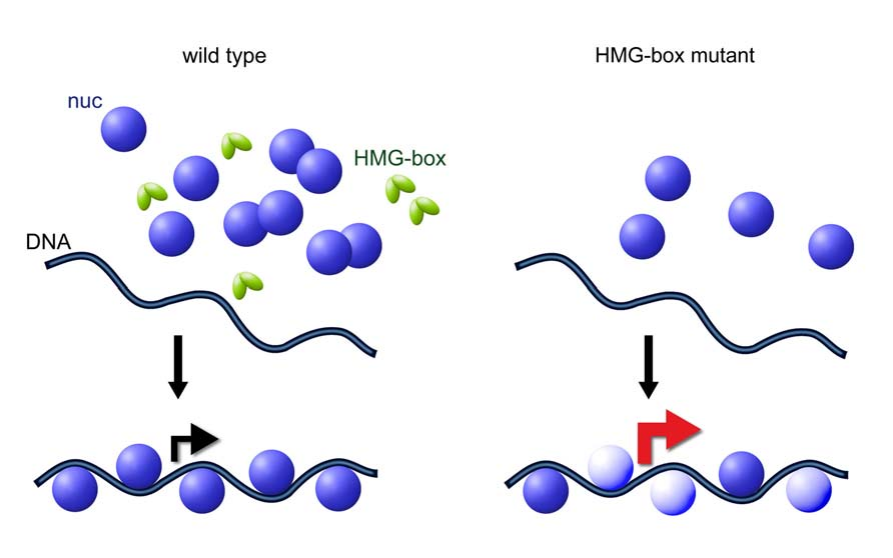
Living with fewer nucleosomes: transcriptional programs are affected when, on some sites, nucleosomes are present only part-time.

The genome of every human cell comprises a length of about two meters of DNA. To pack this very long, thin molecule into the microscopic cell nucleus, it must be spooled up very tightly. In the first step of compaction, the DNA molecules wrap around histone proteins to produce nucleosomes—the dense, bead-like structures commonly thought of as the basic unit of genome packaging. It's no surprise that this packaging affects the accessibility of the DNA and how it functions. Nucleosomes not only protect the fragile DNA molecules from damage, but also limit the access of other proteins to the DNA, regulating functions like gene transcription. Cells that lack proteins required for histone formation have been shown to be susceptible to genetic damage and early death.

New research by Barbara Celona and colleagues at the San Raffaele Scientific Institute in Milan, Italy, shows that when the cell has fewer nucleosomes than usual it affects some genes more than others. The findings run counter to the model that a decreased nucleosome count would be felt equally throughout the genome: in fact, parts of the DNA that normally have high nucleosome concentrations still have high concentrations when the total nucleosome count drops, whereas sites that normally have lower nucleosome counts tend to lose more of their nucleosomes. The researchers also document the effects of having fewer histones than normal: although yeast and mammalian cells with low histone counts are viable, they are more susceptible to genetic damage and show increased rates of transcription for some genes.

To explore the effect of a lower nucleosome count, the researchers worked with mammalian cells lacking the gene for HMGB1, a high mobility group protein that they showed to facilitate nucleosome assembly, and yeast cells lacking the gene that encodes two Nph6 proteins (Nph6a and Nph6b), which are believed to be functionally equivalent to mammalian HMGB1 proteins. (Yeast cells lacking Nph6a/b proteins are viable but defective.)

Before analyzing nucleosome formation directly, the researchers first examined whether the mutant cells were more susceptible to genetic damage. Previous studies had shown that UV radiation damaged HMGB1/Nph6-negative mutants more easily than normal, wild-type cells. For this study, the researchers subjected yeast and mammalian cells to ionizing radiation. The mutants had more DNA breaks than their wild-type counterparts, indicating that the DNA in the mutant cells is more accessible to radiation.

To confirm that the HMGB1 proteins affect histone content, the team worked with mouse and human cells. Upon examining the proteins bound to DNA in HMGB1-negative cells, they found that the four core histone proteins were reduced by about 20% compared to wild-type cells. To confirm that their results were not an artifact of cells in culture, they also showed that HMGB1-negative mouse liver cells contained about 20% fewer histones. In yeast cells lacking Nph6 the story was similar. The mutant yeast and mammalian cells also had fewer nucleosomes.

The position of nucleosomes in the genome is important for how they protect DNA and control transcription, yet scientists are still unsure why nucleosomes form where they do. One hypothesis holds that nucleosomes are positioned between “barriers”, and when there are fewer nucleosomes than normal the remaining ones will shift positions, spreading out between the barriers. Another theory argues that nucleosome location is determined by DNA sequences, and that some sequences have a greater affinity for nucleosomes than others. According to this second theory, when there are fewer nucleosomes, their position should not change, but some sequences will lose “their” nucleosomes more often (decreased occupancy).

Using a variety of techniques, the researchers compared nucleosome positioning in wild-type and Nhp6-negative yeast cells and found that in the absence of Nhp6, nucleosome “occupancy” on the DNA changed, but not uniformly. Some sites appear to have similar nucleosome occupancy in wild-type cells, whereas in the rest of the DNA the nucleosome occupancy is clearly reduced. The genes that suffered the most nucleosome loss were those that had lowest levels of nucleosome occupancy to start with. In mammalian cells, the researchers did not assess nucleosome positioning directly but noted that nucleosome spacing appears relatively conserved in HMGB1-negative cells, which would not be expected if nucleosomes were redistributed according to the first hypothesis.

Finally, the researchers examined gene transcription in mammalian cells—the process of converting the DNA code into products that function in the cell. Because nucleosomes help regulate access to DNA, fewer nucleosomes should lead to increased DNA transcription. Sure enough, in HMGB1-negative human cells, which have 20% fewer histones than wild-type cells, the mRNA produced by transcription increased by about 30%. This increase was not uniform, however. About 13% of mRNAs were upregulated either significantly more or less than the majority, indicating that nucleosome depletion does not affect all genes equally. These findings, say the researchers, suggest a new layer of regulation of gene transcription orchestrated by histone availability.

**Celona B, Weiner A, Di Felice F, Mancuso FM, Cesarini E, et al. (2011) Substantial Histone Reduction Modulates Genomewide Nucleosomal Occupancy and Global Transcriptional Output. doi:10.1371/journal.pbio.1001086**

